# Higher doses of loop diuretics limit uptitration of angiotensin-converting enzyme inhibitors in patients with heart failure and reduced ejection fraction

**DOI:** 10.1007/s00392-020-01598-w

**Published:** 2020-01-30

**Authors:** Jozine M. ter Maaten, Pieter Martens, Kevin Damman, Kenneth Dickstein, Piotr Ponikowski, Chim C. Lang, Leong L. Ng, Stefan D. Anker, Nilesh J. Samani, Gerasimos Filippatos, John G. Cleland, Faiez Zannad, Hans L. Hillege, Dirk J. van Veldhuisen, Marco Metra, Adriaan A. Voors, Wilfried Mullens

**Affiliations:** 1 Department of Cardiology, University Medical Centre Groningen, University of Groningen, Hanzeplein 1, 9713 GZ Groningen, The Netherlands; 2 Ziekenhuis Oost-Limburg, Genk, Belgium; 3 University of Bergen, Bergen, Norway; 4 Stavanger University Hospital, Stavanger, Norway; 5 Department of Heart Diseases, Poland and Cardiology Department, Military Hospital, Wroclaw Medical University, Wrocław, Poland; 6 Division of Molecular and Clinical Medicine, School of Medicine, Ninewells Hospital and Medical School, University of Dundee, Dundee, UK; 7 Department of Cardiovascular Sciences, University of Leicester, Leicester, UK; 8 NIHR Leicester Biomedical Research Centre, Glenfield Hospital, Leicester, LE3 9QP UK; 9 Department of Cardiology (CVK), and Berlin-Brandenburg Center for Regenerative Therapies (BCRT), German Centre for Cardiovascular Research (DZHK) Partner Site Berlin, Charité Universitätsmedizin Berlin, Berlin, Germany; 10 Department of Cardiology and Pneumology, University Medical Center Göttingen (UMG), Göttingen, Germany; 11 Heart Failure Unit, Department of Cardiology, School of Medicine, Athens University Hospital Attikon, National and Kapodistrian University of Athens, Athens, Greece; 12 School of Medicine, University of Cyprus, Nicosia, Cyprus; 13 National Heart and Lung Institute, Royal Brompton and Harefield Hospitals, Imperial College, London, UK; 14 INSERM, Centre D’Investigations Cliniques Plurithématique 1433, Université de Lorraine, CHRU de Nancy and F-CRIN INI-CRCT, Nancy, France; 15 Department of Medical and Surgical Specialties, Radiological Sciences and Public Health, Institute of Cardiology, University of Brescia, Brescia, Italy

**Keywords:** Heart failure, Loop diuretics, Guideline recommended treatment, ACEi/ARB

## Abstract

**Background:**

Loop diuretics are frequently prescribed to patients with heart failure and reduced ejection fraction (HFrEF) for the treatment of congestion; however, they might hamper uptitration of inhibitors of the renin–angiotensin system.

**Methods:**

Loop diuretic dose at baseline was recorded in 2338 patients with HFrEF enrolled in BIOSTAT-CHF, an international study of HF patients on loop diuretic therapy who were eligible for uptitration of angiotensin-converting enzyme inhibitors (ACEi)/mineralocorticoid receptor antagonists (MRA). The association between loop diuretic dose and uptitration of ACEi/MRA to percentage of target dose was adjusted for a previously published model for likelihood of uptitration and a propensity score.

**Results:**

Baseline median loop diuretic dose was 40 [40–100] mg of furosemide or equivalent. Higher doses of loop diuretics were associated with higher NYHA class and higher levels of NT-proBNP, more severe signs and symptoms of congestion, more frequent MRA use, and lower doses of ACEi reached at 3 and 9 months (all *P* < 0.01). After propensity adjustment, higher doses of loop diuretics remained significantly associated with poorer uptitration of ACEi (Beta per log doubling of loop diuretic dose: − 1.66, *P* = 0.021), but not with uptitration of MRAs (*P* = 0.758). Higher doses of loop diuretics were independently associated with an increased risk of all-cause mortality or HF hospitalization [HR per doubling of loop diuretic dose: 1.06 (1.01–1.12), *P* = 0.021].

**Conclusions:**

Higher doses of loop diuretics limited uptitration of ACEi in patients with HFrEF and were associated with a higher risk of death and/or HF hospitalization, independent of their lower likelihood of uptitration and higher baseline risk.

**Graphic abstract:**

This figure was created with images adapted from Servier Medical Art licensed under a Creative Commons Attribution 3.0
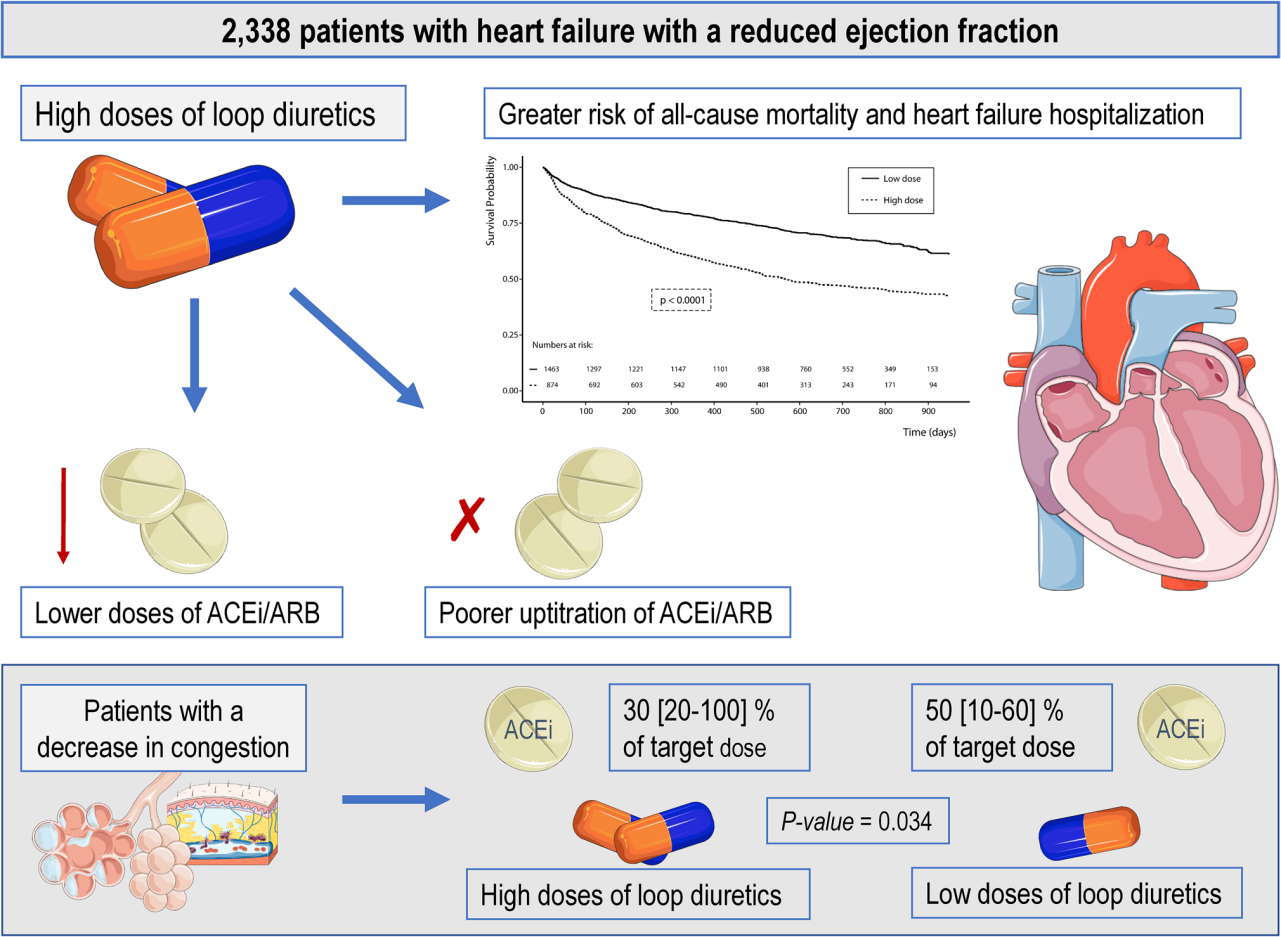

**Electronic supplementary material:**

The online version of this article (10.1007/s00392-020-01598-w) contains supplementary material, which is available to authorized users.

## Introduction

In patients with heart failure, administration of loop diuretics is the cornerstone of the treatment of signs and symptoms of congestion. While loop diuretics are almost ubiquitously used in hospitalized heart failure, data from registries and randomized-controlled trials show that approximately 75–92% of patients with stable heart failure also use loop diuretics chronically [1–4]. Heart failure guidelines recommend to use loop diuretics to reduce the signs and symptoms of congestion, and to use the lowest achievable dose to reach and maintain euvolemia [5]. If patients are asymptomatic, the use of a loop diuretic could be discontinued as loop diuretic downtitration or even withdrawal might be feasible in up to 60% of (selected) stable heart failure patients [6–8]. Such downtitration of loop diuretics might be important as overzealous use of diuretics can result in worsening of renal function, contraction of plasma volume, and lower blood pressures [4, 6]. Additionally, consequent hypovolaemia and hyponatraemia cause increased renin release through its effects on the macula densa and baroreceptors. These detrimental effects of inappropriate use of loop diuretics could also hamper the optimal uptitration of guideline recommended doses of angiotensin-converting enzyme inhibitors (ACEi)/angiotensin receptor blockers (ARB), and mineralocorticoid receptor antagonists (MRA). Observational data illustrate that only a minority of patients are able to attain these target doses of neurohormonal blockers [9, 10]. Therefore, reasons for not uptitrating renin-angiotensin-aldosterone system (RAAS) blockers to the recommended doses should be further explored. We hypothesize that higher doses of loop diuretics might hamper the uptitration of RAAS blockers. We, therefore, aimed to assess the effect of loop diuretic dosage on the ability to uptitrate patients to guideline recommended doses of ACEi/ARB and MRA, as well as to assess the association of loop diuretic dosage with outcome.

## Methods

### Study population

The study design of ‘A systems BIOlogy Study to Tailored Treatment in Chronic Heart Failure’ (BIOSTAT-CHF) has been described previously [11]. In brief, BIOSTAT-CHF was a multicentre, multinational, prospective observational study, in which 2516 patients with new-set or worsening signs and/or symptoms of heart failure from 11 European countries, who were on suboptimal guideline recommended treatment (i.e.,  ≤ 50% of target doses of ACEi/ARBs and beta-blockers), were enrolled. Physicians could enrol patients if they anticipated uptitration or initiation of ACEi/ARB and beta-blockers. Additionally, all patients in BIOSTAT-CHF had to be on a loop diuretic dose equal or more than 40 mg furosemide equivalents at inclusion (40 mg furosemide equals 20 mg torsemide or 1 mg bumetanide). Investigators were encouraged to optimize treatment of heart failure with ACEi/ARB and beta-blockers during the first 3 months of the study, according to the doses indicated in the European Society of Cardiology Guidelines [12]. Patients with HFpEF (162 (6.4%) patients) defined as an ejection fraction > 45% were excluded from the current analyses, as uptitration of guideline directed medical therapy is not always required in these patients.

All patients provided written informed consent to participate in the study and BIOSTAT-CHF was conducted in concordance with the Declaration of Helsinki. The study was approved by national and local ethics committees.

### Study assessments

Both inpatients and outpatients were enrolled, and had a visit at baseline and after 9 months of follow-up. During the first 3 months, the treating physician was encouraged to uptitrated evidence-based therapies to the target doses presented in the 2008 and 2012 ESC heart failure guidelines [12, 13]. The subsequent 6 months were considered as a maintenance phase. Doses of evidence-based therapies were recorded at baseline, 3 months (only for ACEi/ARB and beta-blockers), and 9 months. Target doses of MRAs were based on the doses recommended in the heart failure guidelines, where for both spironolactone and eplerenone, a dose of 50 mg daily is considered target dose [5]. The dose of loop diuretics at baseline was available in 2338 of the 2354 patients (99%) with HFrEF enrolled in BIOSTAT-CHF. Diuretics were calculated into furosemide equivalents (40 mg furosemide = 20 mg torsemide = 1 mg bumetanide). Downtitration of loop diuretics was defined as a decrease in loop diuretic dose from baseline to 9 months.

A previously defined congestion score was calculated as the sum of orthopnoea (0–1), JVP (0–1), and oedema depending on the severity (0–0.33–0.67–1), resulting in a maximum score of 3 points [14, 15]. The clinical congestion score at 9 months was available in 1167 (49.9%) patients. Sensitivity analyses were performed with a clinical congestion score where oedema was scored 0–3 points, yielding a maximum score of 5 points.

Routine laboratory and other biomarker assessments were performed at baseline and 9 months, using previously described assays [16, 17]. Worsening renal function was defined as an increase in creatinine of > 0.3 mg/dL from baseline to 9 months.

The endpoints selected for these analyses were all-cause mortality and the combined endpoint of all-cause mortality or first occurrence of heart failure (HF) hospitalization. HF hospitalization was defined as hospitalization lasting longer than 1 day for which the primary reason was worsening of signs or symptoms of HF, requiring intravenous medications or an increased dose of oral diuretics.

### Statistical analyses

Baseline clinical variables and biomarkers were evaluated over quartiles of loop diuretic dosage. Frequency (percentage) was used to summarize categorical variables while normally distributed continuous variables were summarized with mean ± standard deviations (SD) and non-normally distributed continuous variables with median [interquartile range]. Trends over quartiles of loop diuretic dosage were statistically tested with Cochran–Armitage trend test, Jonckheere–Terpstra, or a linear regression model for categorical variables, non-normally distributed continuous variables, and normally distributed continuous variables, respectively. Univariable and multivariable linear regression analysis was performed with log-transformed loop diuretic dosage as a dependent variable. Transformations were checked using multifractional polynomials. Multivariable linear regression analyses, including all variables with *P* < 0.10 in univariable analysis, were constructed via backward elimination and validated using bootstrap re-sampling with 1000 replicates. The model was tested for collinearity and checked by plotting residuals. Logistic regression was used to investigate the association between loop diuretic downtitration and clinical variables as well as study the association between log-transformed loop diuretic dosage and ACEi/ARB, or MRA use, and whether target dose was reached. The association between log-transformed loop diuretic dosage and percentage of target dose was studied using linear regression. A propensity score was determined using multivariable linear regression with loop diuretic dosage as the dependent variable, using the above described selection and backward elimination. This propensity score reflects the characteristics associated with the prescription of higher doses of loop diuretics. The propensity score included age, hepatomegaly, diastolic blood pressure, previous heart failure hospitalization, history of atrial fibrillation, history of COPD, urea, estimated glomerular filtration rate (eGFR), potassium, N-terminal pro blood natriuretic peptide (NT-proBNP), and plasma renin (Supplementary Table 1). Propensity score adjustment was used to reduce the effect of treatment selection bias in prescribing higher doses of loop diuretic dosage. The association between loop diuretic dose and uptitration of ACEi/ARB and MRAs, was adjusted for three multivariable models. First, we adjusted for age and sex. Second, we performed a multivariable adjustment for a previously published model predicting lower doses of these medications in this cohort [18]. This model included sex, country of inclusion, BMI, and eGFR. Finally, adjustment for a biological plausible model was performed, including age, sex, eGFR, NT-proBNP, and ACEi/ARB use at baseline. Cox proportional hazard regression analysis was performed to examine associations with clinical outcomes. Log-transformed loop diuretic dosage was investigated per doubling. Multivariable models were adjusted for an outcome model specifically developed and validated in the BIOSTAT index and validation cohort [19]. A two-tailed *P* value < 0.05 was considered statistically significant. All analyses were performed using R: A Language and Environment for Statistical Computing, version 3.3.1 (R Foundation for Statistical Computing, Vienna, Austria).

## Results

Median daily loop diuretic dose at baseline was 40 [40–100] mg of furosemide or equivalent. Baseline characteristics over quartiles of loop diuretic dose are presented in Table 1. Patients with higher loop diuretic doses were more frequently hospitalized, had a higher Body Mass Index (BMI), New York Heart Association (NYHA) functional class, as well as more signs and symptoms of congestion, lower blood pressure, and lower left-ventricular ejection fraction. Additionally, higher doses of loop diuretics were associated with poorer renal function, lower albumin, sodium, aldosterone to renin ratio, and higher NT-proBNP levels (all *P* < 0.001).

**Table 1:** Baseline characteristics over quartiles of loop diuretic dose at baseline

	Q1	Q2	Q3	Q4	*P* trend
*N*	1319	120	504	395	
Loop diuretic dose	40 [40–40]	60 [50–60]	80 [80–120]	250 [160–300]	
min–max	1–40	45–60	70–125	130–600	
Demographics					
Sex [% Male(*n*)]	73.6 (971)	76.7 (92)	76 (383)	76.7 (303)	0.147
Age (years)	68.0 ± 12.2	65.9 ± 12.2	69.5 ± 11.8	69.0 ± 11.1	0.005
BMI (kg/m2)	27.6 ± 5.1	27.9 ± 4.8	28.2 ± 6	28.6 ± 6.1	0.002
NYHA class [%(*n*)]					< 0.001
I	3 (39)	3.3 (4)	1.4 (7)	1 (4)	
II	41.8 (551)	31.7 (38)	27.6 (139)	23 (91)	
III	43.2 (570)	41.7 (50)	55.2 (278)	59.2 (234)	
IV	9.5 (125)	19.2 (23)	12.9 (65)	15.2 (60)	
Unknown	2.6 (34)	4.2 (5)	3.0 (15)	1.5 (6)	
LVEF (%)	29.5 ± 7.7	30.7 ± 7.1	28.3 ± 8.2	27.9 ± 8.5	< 0.001
Clinical profile					
Oedema [%(*n*)]	22.5 (239)	24 (24)	35 (151)	41.1 (146)	< 0.001
Oedema above knee (%(n))	5.4 (57)	6.0 (6)	5.4 (23)	11.8 (42)	< 0.001
Orthopnoea [%(*n*)]	29.3 (386)	37.5 (45)	42.7 (215)	40.6 (159)	< 0.001
Rales > 1/3 up lung fields [%(*n*)]	15.2 (87)	10.8 (7)	27.6 (82)	19.7 (46)	0.002
Jugular venous pressure [%(*n*)]	25.9 (216)	34.6 (28)	42 (146)	42.5 (119)	< 0.001
Hepatomegaly [%(*n*)]^a^	9.7 (127)	19.2 (23)	18.7 (94)	23.4 (92)	< 0.001
Third heart tone [%(*n*)]	10.6 (139)	10.8 (13)	9.8 (49)	9.4 (37)	0.463
Systolic blood pressure (mmHg)	126.5 ± 21.9	122.5 ± 20.4	122.3 ± 22.0	119.8 ± 20.4	< 0.001
Diastolic blood pressure (mmHg)	76.8 ± 12.9	74.1 ± 11.8	73.8 ± 14.3	71.6 ± 12.5	< 0.001
Heart rate (beats/min)	79.8 ± 20	79.0 ± 18.3	80.5 ± 18.7	79.6 ± 18	0.823
Hospitalization					
Type of visit [%(*n*)]					< 0.001
Scheduled outpatient	34.7 (458)	23.3 (28)	19.2 (97)	20.3 (80)	
Unscheduled outpatient	6.2 (82)	5 (6)	4.2 (21)	6.3 (25)	
Inpatient hospitalization	59.1 (779)	71.7 (86)	76.6 (386)	73.4 (290)	
Reason for visit [%(*n*)]					0.213
Worsening heart failure	49.5 (653)	49.2 (59)	59.5 (300)	69.6 (275)	
New-onset heart failure	29.6 (390)	28.3 (34)	28.4 (143)	13.9 (55)	
Other reason	20.9 (276)	22.5 (27)	12.1 (61)	16.5 (65)	
Heart failure history					
Years since first diagnosis	1.3 [0.2–6.1]	0.6 [0.3–7.8]	3.2 [0.5–9]	2.9 [0.4–6.6]	0.722
Ischemic heart disease [%(*n*)]	60.8 (709)	59.4 (63)	62 (286)	67 (240)	0.062
Previous HF hospitalization [%(*n*)]	28.7 (378)	32.5 (39)	35.1 (177)	42 (166)	< 0.001
Medical history					
Hypertension [%(*n*)]	59.9 (790)	63.3 (76)	60.5 (305)	66.8 (264)	0.040
Atrial fibrillation [%(*n*)]	41.2 (543)	40 (48)	46.4 (234)	53.2 (210)	< 0.001
Diabetes mellitus [%(*n*)]	28.9 (381)	31.7 (38)	32.9 (166)	44.8 (177)	< 0.001
Laboratory					
Creatinine (umol/L)	96.7 [79–117.3]	100 [79.6–123.8]	109 [91–142.8]	118 [92–158.1]	0.858
Urea (mmol/L)	9.4 [6.8–15.2]	15.4 [11.1–22.9]	11.9 [7.7–19.5]	14.4 [9.7–24.1]	< 0.001
eGFR (ml/min/1.73m^2^)	64.7 [49.1–82.8]	62.3 [47–81]	55.1 [40.7–72.5]	50.5 [34–69.7]	< 0.001
Sodium (mmol/L)	140 [137–142]	139 [137–142]	139 [136–141]	139 [136.8–141]	< 0.001
Potassium (mmol/L)	4.3 [4–4.6]	4.2 [3.9–4.5]	4.2 [3.9–4.5]	4.1 [3.8–4.5]	< 0.001
Albumin (g/L)	33 [28–39]	33 [28–37.5]	32 [26–37]	32 [26–37]	< 0.001
Aldosterone (pg/mL)	94 [46–189]	86.5 [33–166.8]	110 [44.1–231]	97 [44–233]	0.002
Renin (UI/mL)	65.2 [23.4–194.5]	124.1 [36.9–300.1]	127.1 [45.5–368.6]	175.7 [60.5–483.5]	< 0.001
Aldosterone-to-renin ratio	1.5 [0.4–4]	0.7 [0.2–2.4]	0.7 [0.2–2.1]	0.6 [0.1–1.7]	0.001
NT-proBNP (pg/mL)	2211.5 [974.2–4773.2]	2118.5 [1058–4323.2]	3315 [1526–7397.5]	3839.5 [1592–8886.5]	< 0.001

*BMI* body mass index, *eGFR* estimated glomerular filtration rate, *LVEF* left-ventricular ejection fraction, *NT-proBNP* n terminal pro blood natriuretic peptide, *NYHA* New York Heart Association

^a^Based on physical examination

At 9 months, median loop diuretic dose was 40 [40–80] mg of furosemide or equivalent, with a median decline of 0 [− 40–0] mg. A total of 745 patients (37.2%) had a decrease, and 18.6% (373 patients had an increase in diuretic dose at 9 months. A significant number of patients displayed signs of congestion at 9 months: 18.1% of patients in the highest quartile of loop diuretic dosage had oedema above the knee, 12.3% had an elevated JVP, and 12.2% had orthopnoea (all *P* < 0.02, Supplementary Table 2).

Overall, patients with an increase in diuretic dose over 9 months were comparable to patients with a decrease in diuretic dose (Supplementary Table 3), with no notable differences in baseline clinical presentation, laboratory values, or guideline recommended therapy over time. Predictors of loop diuretic downtitration were higher baseline loop diuretic dose, orthopnoea, lower plasma aldosterone levels, higher urea and eGFR at baseline, and no history of a cardiomyopathy, myocardial infarction, or diabetes mellitus (Table 2). Uptitration of ACEi/ARB or MRA was not independently associated with a decrease in loop diuretic dose at 9 months.

**Table 2:** Multivariable model downtitration of loop diuretics at 9 months

	OR (CI)	*P *value
Loop diuretic dose at baseline	3.45 (2.91–4.12)	< 0.001
Orthopnoea	1.50 (1.18–1.90)	< 0.001
History of cardiomyopathy	0.62 (0.48–0.79)	< 0.001
Myocardial infarction	0.67 (0.52–0.88)	0.002
Diabetes mellitus	0.65 (0.50–0.83)	< 0.001
Aldosterone	0.72 (0.57–0.91)	0.005
Urea	1.01 (100–1.02)	0.038
eGFR	1.01 (1.00–1.01)	0.008

*eGFR* estimated glomerular filtration rate

### Loop diuretic dosage and ACEi/ARB and MRA uptitration

At baseline, there were no differences in dosage of ACEi/ARB; yet, after 3 months of encouraged uptitration and an additional 6 month maintenance phase, patients with higher doses of loop diuretics at baseline were less likely to use ACEi/ARB, and used lower doses both at 3 and 9 months (Tables 3, and 4). In patients with higher doses of loop diuretics, symptoms, side-effects, and non-cardiac organ dysfunction were more frequently noted as the reasons for not achieving target dose of ACEi/ARB (Table 3). After multivariable adjustment for the biological plausible model, as well as after multivariable adjustment for the previously published model for likelihood of uptitration, the association between higher loop diuretic dose and less use/dose of ACEI/ARB remained statistically significant (Table 4).

**Table 3:** Doses of ACEi/ARB and MRA at baseline, 3 months, and 9 months over quartiles of loop diuretic doses at baseline

	Q1	Q2	Q3	Q4	*P *trend
*N*	1319	120	504	395	
Loop diuretic dose	40 [40–40]	60 [50–60]	80 [80–120]	250 [160–300]	
ACE inhibitors or angiotensin receptor blockers					
ACE inhibitors or angiotensin receptor blockers at baseline [%(*n*)]	75.4 (994)	66.7 (80)	70.6 (356)	71.9 (284)	0.045
Target dose at baseline [%(*n*)]	17.7 (176)	21.2 (17)	18 (64)	20.8 (59)	0.328
Percentage of target dose at baseline (%)	20 [0–50]	20 [0–50]	20 [0–50]	20 [0–50]	0.717
ACE inhibitors or angiotensin receptor blockers at 3 months [%(*n*)]	91.8 (1211)	85.8 (103)	84.9 (428)	82.5 (326)	< 0.001
Target dose at 3 months [%(*n*)]	26.2 (317)	30.1 (31)	24.8 (106)	20.6 (67)	0.061
Percentage of target dose at 3 months (%)	50 [20–80]	50 [20–100]	30 [10–60]	30 [10–50]	< 0.001
Change in percentage of target dose from baseline to 3 months (%)^a^	0 [0–25]	0 [0–26.6]	0 [0–25]	0 [0–12.5]	< 0.001
ACE inhibitors or angiotensin receptor blockers at 9 months [%(*n*)]	90.8 (1197)	86.7 (104)	82.9 (418)	78 (308)	< 0.001
Target dose at 9 months [%(*n*)]	29.2 (349)	30.8 (32)	25.6 (107)	21.4 (66)	0.006
Percentage of target dose at 9 months (%)	50 [20–100]	50 [10–70]	20 [10–60]	20 [0–50]	< 0.001
Change in percentage of target dose from baseline to 9 months (%)^a^	0 [0–25]	0 [0–50]	0 [0–25]	0 [0–12.5]	< 0.001
Reasons for not uptitrating ACE inhibitors [%(*n*)]					< 0.001
Symptoms	7.2 (95)	5.0 (6)	9.3 (47)	10.4 (41)	
Side-effects	9.2 (122)	8.3 (10)	17.3 (87)	12.9 (51)	
Non-cardiac organ dysfunction	2.4 (32)	1.7 (2)	2.0 (10)	3.3 (13)	
Other	5.4 (71)	8.3 (10)	6.9 (35)	9.1 (36)	
Uptitrated according to guidelines	53.8 (709)	50.0 (60)	38.9 (196)	32.2 (127)	
Unknown	22.0 (290)	16.7 (32)	25.6 (129)	32.2 (127)	
Mineralocorticoid antagonists					
MRA at baseline [%(*n*)]	51.4 (678)	58.3 (70)	57.5 (290)	61.0 (241)	< 0.001
Target dose at baseline (%(n))	16.6 (106)	20.8 (11)	23.6 (63)	26.4 (56)	0.001
Percentage of target dose at baseline (%)	50 [50–50]	50 [50–50]	50 [50–50]	50 [50–100]	0.001
MRA at 9 months [%(*n*)]	58.1 (653)	61.2 (60)	64.2 (250)	61.4 (173)	0.040
Target dose at 9 months [%(*n*)]	16.0 (114)	12.5 (7)	16.7 (45)	22.8 (43)	0.071
Percentage of target dose at 9 months [%(*n*)]	50 [25–50]	50 [18.8–50]	50 [25–50]	50 [25–50]	0.450
Change in percentage dose from baseline to 9 months (%)^a^	0 [-100–0]	0 [-100–0]	0 [-76.2–0]	0 [-100–0]	0.861

*ACEi/ARB* angiotensin-converting enzyme inhibitors/angiotensin receptor blockers, *MRA* mineralocorticoid antagonists

^a^Defined as: percentage of target dose at 3 months minus percentages of target dose at baseline divided by percentage of target dose at baseline times 100

**Table 4:** Loop diuretic dose and ACEi/ARB over time

	ACEi/ARB use at 3 months		Target dose at 3 months		Change in percentage of target dose from baseline to 3 months		Change in percentage of target dose from baseline to 9 months	
	OR (CI)	*P* value	OR (CI)	*P* value	Beta (CI)	*P* value	Beta (CI)	*P* value
Log loop diuretic dose (per doubling)								
Univariable	0.78 (0.70–0.86)	< 0.001	0.88 (0.80–0.95)	0.003	− 2.81 (− 3.89 to 1.72)	< 0.001	− 4.04 (− 5.34 to 2.72)	< 0.001
Multivariable^a^	0.78 (0.71–0.87)	< 0.001	0.88 (0.81–0.96)	0.004	− 2.70 (− 3.78 to 1.62)	< 0.001	− 3.87 (− 5.18 to 2.57)	< 0.001
Multivariable^b^	0.88 (0.79–0.99)	0.028	0.85 (0.75–0.97)	0.013	− 1.93 (− 3.06 to 0.81)	< 0.001	− 2.73 (− 4.04 to 1.42)	< 0.001
Multivariable^c^	0.88 (0.78–0.99)	0.031	0.83 (0.74–0.94)	0.004	− 2.34 (− 3.49 to 1.20)	< 0.001	− 3.43 (− 4.77 to 2.09)	< 0.001
Propensity score adjusted	0.93 (0.82–1.05)	0.260	0.96 (0.85–1.09)	0.557	− 1.66 (− 3.07 to 0.25)	0.021	− 2.09 (− 3.74 to 0.44)	0.013

*ACEi/ARB* angiotensin-converting enzyme inhibitor/angiotensin receptor blocker, *AF* alkaline phosphatase, *BMI* body mass index, *CI* confidence interval, *DBP* diastolic blood pressure, *eGFR* estimated glomerular filtration rate, *HR* heart rate, *NT-proBNP* n terminal pro blood natriuretic peptide

^a^Adjusted for age and sex

^b^Adjusted for sex, country, BMI, AF, and eGFR

^c^Adjusted for log NT-proBNP, eGFR, age, sex, and ACE/ARB use at baseline

Additionally, higher doses of loop diuretics at baseline were significantly associated with smaller increases in percentage of target doses of ACEi/ARB in univariable and multivariable analyses (Table 4). This association remained significant after propensity adjustment, i.e., higher doses of loop diuretics remained significantly associated with less uptitration both from baseline to 3 months (*P* = 0.021), and from baseline to 9 months (*P* = 0.013).

At baseline, patients in the highest quartile of loop diuretic dose at baseline were more likely to use MRAs and used higher doses (Table 3). At 9 months, there were, however, no significant differences in (change in) percentage of target doses of MRAs (Tables 3, 5).

**Table 5:** Loop diuretic dose and MRA over time

	MRA use at 9 months		Target dose at 9 months		Change in percentage of target dose from baseline to 3 months		Change in percentage of target dose from baseline to 9 months	
	OR (CI)	*P* value	OR (CI)	*P* value	Beta (CI)	*P* value	Beta (CI)	*P* value
Log loop diuretic dose (per doubling)								
Univariable	1.04 (0.96–1.13)	0.350	1.16 (1.01–1.33)	0.031	NA	NA	0.53 (− 3.42 to 4.47)	0.794
Multivariable^a^	1.05 (0.97–1.15)	0.249	1.18 (1.03–1.35)	0.017	NA	NA	0.80 (− 3.15 to 4.75)	0.692
Multivariable^b^	1.10 (0.93–1.32)	0.274	1.24 (1.03–1.50)	0.027	NA	NA	4.24 (− 0.07 to 8.54)	0.054
Multivariable^c^	1.09 (0.92–1.30)	0.333	1.15 (0.96–1.37)	0.121	NA	NA	2.22 (− 2.00 to 6.45)	0.301
Propensity score adjusted	1.05 (0.94–1.17)	0.370	1.14 (0.97–1.35)	0.117	NA	NA	0.79 (− 4.27 to 5.86)	0.758

*AF* alkaline phosphatase, *BMI* body mass index, *CI* confidence interval, *DBP* diastolic blood pressure, *eGFR* estimated glomerular filtration rate, *HR* heart rate, *MRA* mineralocorticoid receptor antagonist, *NT-proBNP* n terminal pro blood natriuretic peptide

^a^Adjusted for age, and sex

^b^Adjusted for sex, country, BMI, AF, and eGFR

^c^Adjusted for log NT-proBNP, eGFR, age, sex, and ACE/ARB use at baseline

There was no significant interaction between loop diuretic dosage and site of enrolment on successful uptitration, nor between worsening/new-onset heart failure or in-/outpatients and loop diuretic dosage. Additionally, there was no significant association between loop diuretic doses and uptitration of beta-blockers after propensity adjustment (Supplementary Tables 4 and 5).

### Loop diuretic dosage and congestion

As higher doses of loop diuretics are most frequently driven by signs and symptoms of congestion, we assessed the impact of congestion on loop diuretic dosing and (successful) uptitration of ACEi/ARBs. Patients with a higher congestion score at baseline were more likely to receive higher doses of loop diuretics at baseline as well as at 9 months, and used a significantly lower percentage of target dose of ACEI/ARB at baseline and at subsequent time points (Supplementary Table 6). At 9 months, 846 (72.5%) patients were judged euvolemic based on the clinical congestion score of which 313 (37.0%) patients received uptitration of ACEi/ARB, and 305 (36.1%) patients were downtitrated in terms of loop diuretic dose. Of the 321 (27.5%) patients that displayed signs and symptoms of congestion at 9 months, 106 (33.0%) patients received uptitration of ACEi/ARB, and 94 (29.3%) patients were downtitrated in terms of loop diuretic dose.

**Fig. 1:**
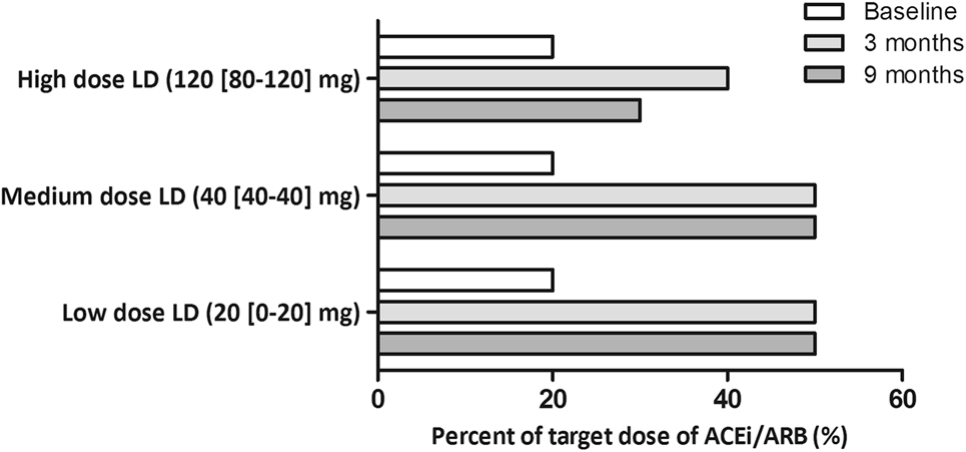
Median percentage of target dose of ACEi/ARB during follow-up in patients with a decrease in congestion score subdivided based on loop diuretic dosage at 9 months. *ACEi/ARB* angiotensin-converting enzyme inhibitors/angiotensin receptor blockers, *LD* loop diuretics

To elucidate the association between loop diuretic dosing, congestion, and uptitration of ACEi/ARB, we divided patients based on a change in congestion score (decrease versus no change/increase) and the dose of loop diuretics at 9 months (Supplementary Table 7). Patients with a decrease in congestion score but persistent high doses of loop diuretics at 9 months were less likely to receive higher percentage of target doses of ACEi/ARB at 9 months (*P* = 0.034), and were less well uptitrated compared to patients with no change/increase in clinical congestion score and low/medium doses of loop diuretics (Fig. 1), underscoring the relation between high doses of loop diuretics and inability to uptitrate ACEi/ARB. Sensitivity analyses with a congestion score attributing greater value to oedema yielded similar findings.

### Loop diuretic dosage and outcomes

**Fig. 2:**
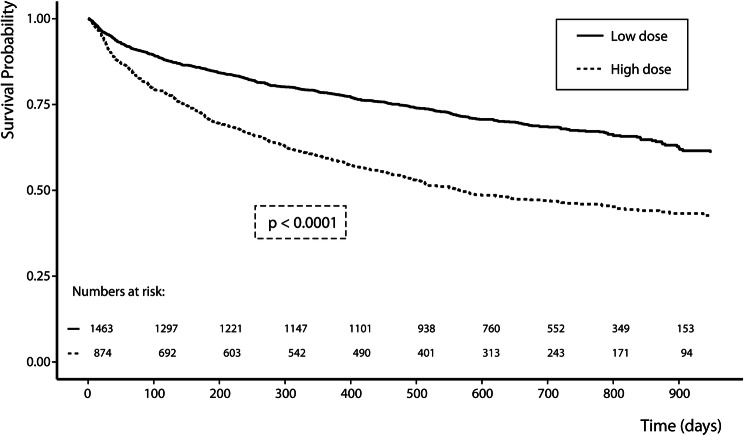
Kaplan–Meier combined endpoint of all-cause mortality and heart failure hospitalization for loop diuretic dosing (low vs. high: > 80 mg of furosemide)

**Fig. 3:**
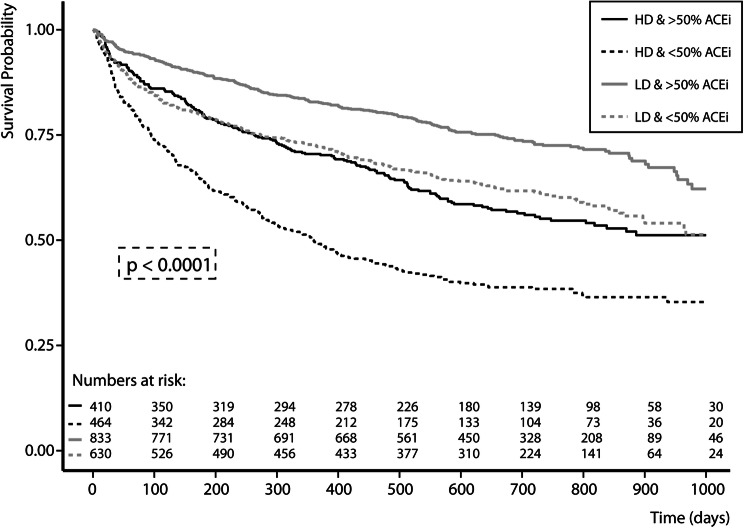
Kaplan–Meier combined endpoint of all-cause mortality and heart failure hospitalization for baseline loop diuretic dosing and > 50% of target dose of ACEi/ARB at 3 months. *ACEi/ARB* angiotensin-converting enzyme inhibitors/angiotensin receptor blockers, *HD* high-dose loop diuretics, *LD* low-dose loop diuretics

During a median follow-up of 21 [16–27] months, 602 (25.7%) patients died, 567 (27.9%) patients were hospitalized for heart failure, and 939 (40.2%) patients experienced the combined endpoint. Higher doses of loop diuretics were independently associated with an increased risk of the combined endpoint of all-cause mortality and heart failure hospitalization [HR per doubling of loop diuretic dosage: 1.06 (1.01–1.12), *P* = 0.021]. Kaplan–Meier curves for the combined endpoint for high (> 80 mg of furosemide or equivalent) versus low dose of loop diuretics are shown in Fig. 2, illustrating a higher risk with a higher loop diuretic dose (log rank, *P* < 0.001). These results remain significant after multivariable adjustment (Supplementary Table 8). In patients with a high dose of loop diuretics (> 80 mg furosemide or equivalent), treatment with > 50% of target dose of ACEi/ARB at 3 months was associated with a significantly lower risk of the combined endpoint (Fig. 3, log-rank *P* < 0.001, Supplementary Table 8) compared to patients who were treated with ≤ 50% of the target dose. Even though patients with an increase in loop diuretic dose experienced numerically more events compared to patients with a decrease [182 (48.8%) versus 234 (31.4%) events, *P* < 0.001], change in loop diuretic dose over time was not independently associated with an increased risk of the combined endpoint [HR per doubling of change in loop diuretic dose censored at 9 months: 0.92 (0.38–2.20), *P* = 0.842].

Higher doses of loop diuretics are independently associated with an increased risk of worsening renal function, even after adjustment for the propensity score and baseline creatinine [OR per doubling of loop diuretic dosage: 1.33 (1.15–1.55), *P* < 0.001]. *Change* in loop diuretic dose was not associated with worsening renal function.

## Discussion

This study provides novel and clinically relevant information regarding the impact of loop diuretic dosage on uptitration of RAAS blockers in patients with heart failure and a reduced ejection fraction. First, in accordance with previous studies, higher doses of loop diuretics at baseline are associated with more severe heart failure, more congestion, worsening renal function, and worse outcomes. Second, there is a significant association between higher loop diuretic dosage and less successful uptitration of guideline recommended doses of ACEi/ARB, but not of MRA or beta-blockers. Third, our data suggest that the association between loop diuretics and uptitration of ACEi/ARB is only partly driven by factors influencing the prescription of higher doses of loop diuretics, as the association between uptitration of guideline recommended ACEi/ARB treatment and loop diuretic dosage remained significant even after propensity score adjustment. Fourth, in patients with an improvement in congestion yet persistent high doses of loop diuretics, uptitration of ACEi/ARB was poorer. These data collectively support the recommendation to dynamically adjust loop diuretic dose to facilitate uptitration of ACEi/ARBs.

### Loop diuretics and guideline recommended treatment with RAAS blockers

Loop diuretics are the first-choice therapy for signs and symptoms of congestion in patients with heart failure, and are used in the majority of heart failure patients [1, 5]. No studies have shown that diuretics decrease mortality risk and several studies have even suggested an association between higher doses of loop diuretics and a higher risk of death, worsening renal function, and symptoms such as hypotension [4, 6]. Higher doses of loop diuretics might also influence the ability to uptitrate doses of RAAS blockers, which we sought to investigate in this study. In clinical practice, not all patients are treated with the recommended doses of neurohormonal blockers. BIOSTAT-CHF was designed to investigate the effects of 3 months of encouraged uptitration of ACEi/ARB and beta-blocker doses on clinical outcomes. In addition, we aimed to study patient profiles associated with impaired uptitration to ultimately move forward to a more personalized treatment approach in treating heart failure patients [11]. By design, this study provided a good context to assess the effect of loop diuretic dosage on successful uptitration of guideline recommended treatment. It should, however, be noted that despite the encouraged uptitration, the number of patients receiving target doses at 3 months did not differ greatly from data from registries [9, 10]. In this study, we showed that patients with higher doses of loop diuretics showed signs of more severe heart failure and congestion. We also found a significant association between loop diuretic dosing and (percentage of) target dose of ACEi/ARB at 3 and 9 months. Interestingly, the association between higher doses of loop diuretics and smaller increases in ACEi/ARB doses over time remained significant after adjustment for the likelihood to be uptitrated, as well as after propensity score adjustment. The persistent significant association, even after propensity score adjustment, suggests that this association is independent of clinical characteristics leading to the prescription of higher doses of loop diuretics. These findings confirm our hypotheses that higher doses of loop diuretics hinder the uptitration of ACEI/ARB in these patients, and that the lower dose of ACEi/ARB attained in the high loop diuretic dose group may not merely be the reflection of sicker patients. Effects of higher loop diuretic doses on hypotension, worsening renal function, and electrolyte imbalances might influence the physician’s decision in not uptitrating ACEi/ARB to guideline recommended doses. Interestingly, we did not observe a significant association between blood pressure and uptitration or downtitration of loop diuretics. Our data suggest that downtitration of loop diuretics to the lowest achievable dose (in euvolemic patients) as recommended by the heart failure guidelines could facilitate a better uptitration of ACEi/ARB.

In contrast, we did not find an association between higher loop diuretic doses and uptitration of MRAs and beta-blockers. A possible explanation for the lack of association with MRA uptitration is that patients with higher doses of loop diuretics at baseline have more severe heart failure and as such more frequently already used MRAs. Furthermore, MRAs are often initiated at target dose; therefore, uptitration is not pursued in clinical practice. Finally, the lack of an association between loop diuretic doses and beta-blocker uptitration might be the result that triggers to limit uptitration of beta-blockers, such as a low heart rate, are not similarly influence by loop diuretic dose, as triggers to limit uptitration of ACEi/ARB (e.g., worsening of renal function and hypotension).

An additional important finding of the present study was that patients with a decrease in signs and symptoms of congestion but residual high doses of loop diuretics were less likely to receive uptitration with ACEi/ARB. Moreover, the doses these patients eventually attained were actually lower than doses observed in patients with persistent congestion, yet with lower doses of loop diuretics. The higher doses of loop diuretics in the group with an improvement of congestion could indicate a phenotype requiring higher doses to maintain euvolemia and as such more severe heart failure, precluding uptitration of neurohormonal blockers. Yet, it could also be hypothesized that these patients could not be uptitrated due to the high doses of loop diuretics, which may not have been necessary based on the congestion status of the patient. Unfortunately, due to the small groups of patients with this data available, further analysis was not able to shed more light on this. Nevertheless, based on our findings and the consensus from a recent position paper, we would advise attempting loop diuretic downtitration in patients without any residual signs and symptoms of congestion (i.e., euvolemic patients), to facilitate successful uptitration of neurohormonal blockers [20].

### Loop diuretics and outcome

Several studies have shown an association between higher doses of loop diuretics and poor outcome [4, 21]. Our study corroborates the previous findings of an association between higher loop diuretic dose and adverse clinical outcome. The finding that loop diuretic downtitration was not associated with improved outcome might be related to the fact that loop diuretic downtitration was particularly possible in patients treated at baseline with higher doses. This might indicate a selection bias towards a sicker patient population, precluding a detection of a beneficial effect of loop diuretic downtitration. Another explanation could be that physicians are generally very good in identifying patients in which diuretics can be downtitrated or even withheld, which is in line with previous findings by Martens et al. [7].

We found a significant association between higher loop diuretic dosage and worsening renal function, which remained statistically significant after propensity adjustment, and was independent of baseline renal function. This detrimental effect of loop diuretics on worsening renal function is, perhaps, directly related to the pharmacology of loop diuretics, since renal blood flow is decreased by loop diuretics through the so-called tubuloglomerular feedback.

### Strengths and limitations

This is the first study to assess the effect of loop diuretic dosage on uptitration of doses of ACEi/ARB. Strengths of the study are the design of the BIOSTAT-CHF trial, making it a suitable cohort to assess this research question, as well as the number of patients enrolled in different European centres. Limitations are the retrospective, observational design, making it impossible to prove causality, and merely allows us to describe associations. Furthermore, propensity score adjustment is in our opinion the best approach to correct for treatment selection bias in prescribing higher doses of loop diuretic dosage; however, we cannot exclude residual confounding. Uptitration was encouraged, yet not forced and left to the discretion of the treating physician. Reasons for not uptitrating guideline-recommended therapies were carefully collected, yet unfortunately often specified as “other”. Reasons for changes in doses of diuretics were not collected. Diuretic doses were relatively low at the start of the study and were only available at time of enrolment and at 9 months. Changes in the meantime, such as during the index hospitalization, were not captured. Signs and symptoms of congestion at 9 months were only available in 49.9% of patients alive at 9 months. Additionally, a limited number of echocardiographic parameters were available, which did not include right-ventricular function or specific assessments of valve dysfunction. Finally, even though uptitration was encouraged, the number of patients in BIOSTAT-CHF that achieved target doses of ACEi/ARB was limited [22].

## Conclusions

In patients with HFrEF, higher doses of loop diuretics are associated with poorer uptitration of ACEi/ARB and with a higher risk of death and/or heart failure hospitalization, independent of the lower likelihood of uptitration and higher baseline risk.

## Electronic supplementary material

Below is the link to the electronic supplementary material.

Supplementary file1 (DOCX 49 kb)

## Abbreviations

ACEi: Angiotensin-converting enzyme inhibitor
ARB: Angiotensin receptor blocker
BIOSTAT-CHF: A systems BIOlogy Study to Tailored Treatment in Chronic Heart Failure
eGFR: Estimated glomerular filtration rate
HF: Heart failure
NT-proBNP: N-terminal pro blood natriuretic peptide
NYHA: New York Heart Association
MRA: Mineralocorticoid receptor antagonists
RAAS: Renin-angiotensin-aldosterone system
