# Supplementary material for: Higher doses of loop diuretics limit uptitration of angiotensin-converting enzyme inhibitors in patients with heart failure and reduced ejection fraction
Source: Clin Res Cardiol. 2020 Jan 30;109(8):1048–59. doi: 10.1007/s00392-020-01598-w (PMC7375987; doi:10.1007/s00392-020-01598-w)
Supplement: Supplementary file 1 — Supplementary file1 (DOCX 49 kb) [file 392_2020_1598_MOESM1_ESM.docx]

**Supplementary material**

**Higher doses of loop diuretics limit uptitration of angiotensin converting enzyme inhibitors in patients with heart failure and reduced ejection fraction**

Jozine M. ter Maaten, Pieter Martens, Kevin Damman, Kenneth Dickstein, Piotr Ponikowski, Chim C. Lang, Leong L. Ng, Stefan D. Anker, Nilesh J. Samani, Gerasimos Filippatos, John G. Cleland, Faiez Zannad, Hans L. Hillege, Dirk J. van Veldhuisen, Marco Metra, Adriaan A. Voors, Wilfried Mullens

***Supplementary table 1 Multivariable regression log loop diuretic dose at baseline***

|  | **Beta coeff** | **95% CI** | **T-value** | ***P-value*** |
| --- | --- | --- | --- | --- |
| **eGFR per SD** | -0.147 | -0.20--0.10 | -5.855 | <0.001 |
| **Renin per SD** | 0.096 | 0.06-0.14 | 4.794 | <0.001 |
| **Potassium per SD** | -0.081 | -0.12--0.04 | -4.247 | <0.001 |
| **NT-proBNP per SD** | 0.082 | 0.04-0.12 | 4.154 | <0.001 |
| **Previous heart failure hospitalization** | 0.165 | 0.08-0.25 | 3.943 | <0.001 |
| **Hepatomegaly** | 0.215 | 0.11-0.35 | 3.883 | <0.001 |
| **Diastolic blood pressure per SD** | -0.060 | -0.10--0.02 | -3.054 | 0.002 |
| **Age per SD** | -0.066 | -0.11--0.02 | -2.938 | 0.003 |
| **COPD** | 0.139 | 0.04-0.24 | 2.726 | 0.006 |
| **Urea per SD** | 0.050 | 0.01-0.09 | 2.324 | 0.020 |
| **Atrial fibrillation** | 0.076 | 0.00-0.15 | 1.906 | 0.057 |

*R^2^=0.144*

***Abbreviations:*** COPD: chronic obstructive pulmonary disease, eGFR: estimated glomerular filtration rate, NT-proBNP: n terminal pro blood natriuretic peptide, SD: standard deviation

***Supplementary table 2. Clinical characteristics at 9 months over quartiles of loop diuretics at baseline***

|  | **Q1** | **Q2** | **Q3** | **Q4** | **P-trend** |
| --- | --- | --- | --- | --- | --- |
| **Loop diuretic dose** | 40 [40-40] | 60 [50-60] | 80 [80-120] | 250 [160-300] |  |
| **Clinical profile at 9 months** |  |  |  |  |  |
| **Oedema above the knee (%(n))** | 6.4 (56) | 4.5 (4) | 10.1 (32) | 18.1 (40) | <0.001 |
| **Rales > 1/3 up lung fields (%(n))** | 5.6 (4) | 0 (0) | 9.8 (4) | 7.1 (3) | 0.541 |
| **Jugular venous pressure (%(n))** | 5.1 (36) | 11.8 (8) | 12.1 (32) | 12.3 (23) | <0.001 |
| **Hepatomegaly (%(n))** | 4.1 (42) | 6.4 (6) | 5.1 (19) | 10.8 (28) | <0.001 |
| **Third heart tone (%(n))** | 4 (41) | 3.2 (3) | 2.7 (10) | 3.4 (9) | 0.386 |
| **Orthopnoea (%(n))** | 8.6 (93) | 5.2 (5) | 12.5 (48) | 12.2 (33) | 0.016 |
| **NYHA class (%(n))** |  |  |  |  | <0.001 |
| **I** | 14.4 (190) | 15 (18) | 11.5 (58) | 8.6 (34) |  |
| **II** | 49.8 (657) | 54.2 (65) | 37.1 (187) | 31.9 (126) |  |
| **III** | 16.1 (212) | 10 (12) | 24.2 (122) | 24.1 (95) |  |
| **IV** | 0.3 (4) | 0.8 (1) | 1.2 (6) | 2 (8) |  |
| **Systolic blood pressure (mmHg)** | 124.5±19.3 | 125.3±20.8 | 125.4±22.4 | 121.0±21.4 | 0.031 |
| **Diastolic blood pressure (mmHg)** | 75.0±11.8 | 75.9±12.0 | 74.1±13.2 | 72.0±11.9 | <0.001 |
| **Heart rate (beats/min)** | 71.9±15.3 | 74.4±16.9 | 72.7±15.7 | 72.9±15.2 | 0.713 |

***Abbreviations:*** NYHA: New York Heart Association

***Supplementary table 3. Baseline characteristic decrease/no change/increase of loop diuretics from baseline to 9 months***

|  | **Decrease in dose of loop diuretic** | **No change in dose of loop diuretic** | **Increase in dose of loop diuretic** | ***P-trend*** |
| --- | --- | --- | --- | --- |
| **N =** | 745 | 885 | 373 |  |
| **Loop diuretic dose change** | -40 [-100--40] | 0 [0-0] | 40 [30-125] |  |
| **Loop diuretic dose at baseline** | 80 [40-150] | 40 [40-40] | 40 [40-80] | <0.001 |
| **Loop diuretic dose at 9 months** | 25 [0-40] | 40 [40-40] | 120 [75-240] | <0.001 |
| **Demographics** |  |  |  |  |
| **Sex (% Male(n))** | 73.2 (545) | 75.8 (671) | 75.3 (281) | 0.319 |
| **Age (years)** | 67.2±12.7 | 67.9±11.9 | 68.9±10.7 | 0.024 |
| **BMI (kg/m2)** | 28.1±6 | 27.9±5 | 28.6±5.6 | 0.110 |
| **Weight (kg)** | 82.5±20.3 | 82.5±17.4 | 83.9±18.3 | 0.221 |
| **NYHA class (%(n))** |  |  |  | 0.829 |
| **I** | 2.4 (18) | 3.3 (29) | 1.3 (5) |  |
| **II** | 32.9 (245) | 45.1 (399) | 27.1 (101) |  |
| **III** | 48.6 (362) | 41.6 (368) | 56.6 (211) |  |
| **IV** | 13.2 (98) | 8.1 (72) | 12.6 (47) |  |
| **LVEF (%)** | 29.3±7.8 | 29.4±7.5 | 28.4±8 | 0.091 |
| **Clinical Profile** |  |  |  |  |
| **Oedema (%(n))** | 30 (187) | 18.5 (132) | 35.5 (111) | 0.703 |
| **Orthopnoea (%(n))** | 37.9 (281) | 25.6 (227) | 38.4 (143) | 0.248 |
| **Rales > 1/3 up lung fields (%(n))** | 21.4 (84) | 15.7 (58) | 18 (37) | 0.178 |
| **Jugular venous pressure (%(n))** | 33.7 (174) | 22.7 (132) | 40.3 (94) | 0.671 |
| **Hepatomegaly (%(n))** | 13.1 (97) | 9.8 (87) | 18.9 (70) | 0.061 |
| **Third heart tone (%(n))** | 9.9 (73) | 8.2 (72) | 12.6 (47) | 0.319 |
| **Systolic Blood Pressure (mmHg)** | 124.4±22.2 | 127.5±20.6 | 122±21.4 | 0.080 |
| **Diastolic Blood Pressure (mmHg)** | 75.6±14.5 | 76.8±12.9 | 74.3±11.8 | 0.126 |
| **Heart Rate (beats/min)** | 81.8±20.4 | 78.2±19.1 | 79.7±17.9 | 0.090 |
| **Hospitalization** |  |  |  |  |
| **Type of visit (%(n))** |  |  |  | 0.009 |
| **Scheduled outpatient** | 22.6 (168) | 39.1 (346) | 25.7 (96) |  |
| **Unscheduled outpatient** | 4.7 (35) | 7.6 (67) | 5.4 (20) |  |
| **Inpatients hospitalization** | 72.8 (542) | 53.3 (472) | 68.9 (257) |  |
| **Reason for visit (%(n))** |  |  |  | 0.154 |
| **Worsening heart failure** | 51.1 (381) | 50.1 (443) | 60.9 (227) |  |
| **New-onset heart failure** | 32.8 (244) | 26.2 (232) | 22.8 (85) |  |
| **Other reason** | 16.1 (120) | 23.7 (210) | 16.4 (61) |  |
| **Heart Failure History** |  |  |  |  |
| **Years since first diagnosis** | 3 [0.5-6.4] | 1.1 [0.3-6.7] | 2 [0.3-6.5] | 0.920 |
| **Ischemic heart disease (%(n))** | 56.7 (368) | 62.3 (497) | 62.3 (210) | 0.045 |
| **Previous HF hospitalization (%(n))** | 30.6 (228) | 27.9 (247) | 37.8 (141) | 0.067 |
| **Medical History** |  |  |  |  |
| **Hypertension (%(n))** | 58.9 (439) | 62.8 (556) | 66 (246) | 0.017 |
| **Atrial fibrillation (%(n))** | 44.2 (329) | 38.5 (341) | 50.4 (188) | 0.252 |
| **Diabetes mellitus (%(n))** | 29.1 (217) | 30.1 (266) | 39.9 (149) | 0.001 |
| **Laboratory** |  |  |  |  |
| **Creatinine (umol/L)** | 100 [84.5-126] | 97 [79.6-119] | 105 [84.5-133.5] | 0.060 |
| **Urea (mmol/L)** | 11.2 [7.5-18.6] | 9.3 [6.7-14.8] | 12.1 [8-18.6] | 0.875 |
| **eGFR (ml/min/1.73m2)** | 61.8 [45.8-78.7] | 64.1 [48.6-83.5] | 58.6 [44.2-76.1] | 0.108 |
| **Sodium (mmol/L)** | 140 [137-142] | 140 [138-142] | 140 [137-142] | 0.854 |
| **Potassium (mmol/L)** | 4.2 [3.9-4.5] | 4.3 [4-4.7] | 4.2 [3.9-4.6] | 0.566 |
| **Albumin (g/L)** | 33 [28-38] | 34 [29-39] | 32 [27-38] | 0.842 |
| **Aldosterone (pg/mL)** | 92.9 [41-187] | 96 [48-200.2] | 102.7 [48.8-217.8] | 0.059 |
| **Renin (UI/mL)** | 88.7 [30.2-249.8] | 72.8 [25.2-204.9] | 118.8 [33.1-303.3] | 0.934 |
| **NT-proBNP (pg/mL)** | 2731 [1200-5923] | 1942 [789.3-4143] | 2918 [1442.5-6313.5] | 0.853 |
| **Clinical characteristics at 9 months** |  |  |  |  |
| **Oedema above the knee (%(n))** | 8 (40) | 6.7 (47) | 15 (45) | 0.005 |
| **Rales > 1/3 up lung fields (%(n))** | 11.3 (6) | 4.3 (3) | 4.7 (2) | 0.170 |
| **Jugular venous pressure (%(n))** | 7.1 (30) | 4.8 (27) | 17.1 (42) | <0.001 |
| **Hepatomegaly (%(n))** | 3.6 (21) | 4.7 (39) | 10.1 (35) | <0.001 |
| **Third heart tone (%(n))** | 2.2 (13) | 3.3 (27) | 6.6 (23) | 0.001 |
| **Orthopnoea (%(n))** | 8.3 (51) | 8.6 (73) | 15.2 (55) | 0.002 |
| **NYHA class (%(n))** |  |  |  | <0.001 |
| **I** | 19.5 (145) | 14.5 (128) | 7.2 (27) |  |
| **II** | 42.3 (315) | 60.5 (535) | 49.3 (184) |  |
| **III** | 17.3 (129) | 19.2 (170) | 38.1 (142) |  |
| **IV** | 1.3 (10) | 0.9 (8) | 0.3 (1) |  |
| **Systolic blood pressure (mmHg)** | 125.7±21.5 | 124.8±19.5 | 120.4±20.1 | <0.001 |
| **Diastolic blood pressure (mmHg)** | 75.0±12.8 | 74.7±11.9 | 72.8±11.6 | 0.008 |
| **Heart rate (beats/min)** | 72.2±15.6 | 711.6±14.8 | 74.6±16.5 | 0.018 |

***Abbreviations:*** BMI: body mass index, eGFR: estimated glomerular filtration rate, LVEF: left ventricular ejection fraction, NT-proBNP: n terminal pro blood natriuretic peptide, NYHA: New York Heart Association

***Supplementary table 4: Doses of beta-blockers at baseline, 3 months and 9 months over quartiles of loop diuretic doses at baseline***

|  | **Q1** | **Q2** | **Q3** | **Q4** | ***P-trend*** |
| --- | --- | --- | --- | --- | --- |
| **N =** | 1319 | 120 | 504 | 395 |  |
| **Loop diuretic dose** | 40 [40-40] | 60 [50-60] | 80 [80-120] | 250 [160-300] |  |
| **Beta-blockers** |  |  |  |  |  |
| **Beta-blockers at baseline (%(n))** | 84.5 (1115) | 84.2(101) | 81.3 (410) | 85.3(337) | 0.640 |
| Target dose at baseline (%(n)) | 5.8 (77) | 3.3 (4) | 5.4 (27) | 4.8 (19) | 0.421 |
| Percentage of target dose at baseline (%) | 20 [10-50] | 10 [10-20] | 10 [0-30] | 20 [10-50] | 0.560 |
| **Beta-blockers at 3 months (%(n))** | 93.2 (1229) | 94.2 (113) | 90.9 (458) | 88.9 (351) | 0.004 |
| Target dose at 3 months (%(n)) | 14.0 (185) | 16.7 (20) | 10.3 (52) | 9.9 (39) | 0.008 |
| Percentage of target dose at 3 months (%) | 20 [10-50] | 20 [10-50] | 20 [10-50] | 20 [10-50] | 0.063 |
| Change in percentage of target dose from baseline to 3 months (%)^#^ | 0 [0-16.7] | 4.2 [0-25] | 0 [0-16.7] | 0 [0-12.5] | 0.056 |
| **Beta-blockers at 9 months (%(n))** | 93.5 (1233) | 92.5 (111) | 90.1 (454) | 87.6 (346) | <0.001 |
| Target dose at 9 months (%(n)) | 16.5 (217) | 20.8 (25) | 11.1 (56) | 11.9 (47) | 0.002 |
| Percentage of target dose at 9 months (%) | 20 [10-50] | 20 [10-50] | 20 [10-50] | 20 [10-50] | 0.001 |
| Change in percentage of target dose from baseline to 9 months (%)^#^ | 0 [0-25] | 8.3 [0-25] | 0 [0-21] | 0 [0-12.5] | 0.001 |

^#^Defined as: percentage of target dose at 3 months minus percentages of target dose at baseline divided by percentage of target dose at baseline times 100

***Supplementary table 5: Loop diuretic dose and beta-blockers over time***

|  | **Beta-blocker use at 3 months** |  | **Target dose at 3 months** |  |  | **Change in percentage of target dose from baseline to 3 months** |  | **Change in percentage of target dose from baseline to 9 months** |  |
| --- | --- | --- | --- | --- | --- | --- | --- | --- | --- |
| **Log loop diuretic dose (per doubling)** | OR (CI) | P-value | OR (CI) | P-value |  | Beta (CI) | P-value | Beta (CI) | P-value |
| **Univariable** | 0.86 (0.77-0.98) | 0.020 | 0.87 (0.78-0.97) | 0.015 |  | -0.77 (-1.54 - -0.02) | 0.044 | -1.86 (-2.79- -0.92) | <0.001 |
| **Multivariable^^^** | 0.87 (0.77-0.99) | 0.027 | 0.88 (0.79-0.98) | 0.019 |  | -0.70 (-1.46 - 0.05) | 0.068 | -1.77 (-2.70 - -0.84) | <0.001 |
| **Multivariable^#^** | 0.88 (0.78-1.00) | 0.059 | 0.84 (0.73-0.97) | 0.017 |  | -0.83 (-1.62 - -0.04) | 0.041 | -1.97 (-2.95 - -0.99) | <0.001 |
| **Multivariable*** | 0.80 (0.68-0.93) | 0.003 | 0.88 (0.76-1.01) | 0.069 |  | -0.70 (-1.51 - 0.11) | 0.089 | -1.60 (-2.59- -0.60) | 0.002 |
| **Propensity score adjusted** | 0.89 (0.77-1.03) | 0.111 | 0.88 (0.76-1.01) | 0.069 |  | -0.15 (-1.13 - 0.82) | 0.755 | -0.59 (-1.76 - 0.57) | 0.318 |

^#^ adjusted for sex, country, BMI, AF, and eGFR

* adjusted for log NTproBNP, eGFR, age, sex, and beta-blocker use at baseline

***Abbreviations:*** AF: alkaline phosphatase, BMI: body mass index, CI: confidence interval, DBP: diastolic blood pressure, eGFR: estimated glomerular filtration rate, HR: heart rate, NT-proBNP: n terminal pro blood natriuretic peptide

***Supplementary table 6: Baseline characteristics over tertiles of congestion score at baseline***

|  | **T1** | **T2** | **T3** | ***P-trend*** |
| --- | --- | --- | --- | --- |
| **N =** | 468 | 542 | 409 |  |
| **Congestion score** | 0 [0-0] | 1 [0.3-1.3] | 2.3 [1.67-2.67] |  |
| **Demographics** |  |  |  |  |
| **Sex (% Male(n))** | 78 (365) | 76.8 (416) | 73.8 (302) | 0.153 |
| **Age (years)** | 66.6±11.8 | 68.1±11.2 | 69.9±11.7 | <0.001 |
| **NYHA class (%(n))** |  |  |  | <0.001 |
| **I** | 3 (14) | 0.9 (5) | 0.5 (2) |  |
| **II** | 61.5 (288) | 32.1 (174) | 7.8 (32) |  |
| **III** | 33.1 (155) | 57.2 (310) | 62.8 (257) |  |
| **IV** | 1.3 (6) | 9 (49) | 26.2 (107) |  |
| **LVEF (%)** | 29.7±7.2 | 28.2±8.2 | 28.3±8.3 | 0.014 |
| **Clinical Profile** |  |  |  |  |
| **Oedema (%(n))** | 0 (0) | 14.4 (78) | 68.5 (280) | <0.001 |
| **Orthopnoea (%(n))** | 0 (0) | 32.5 (176) | 77.5 (317) | <0.001 |
| **Rales > 1/3 up lung fields (%(n))** | 12.1 (15) | 15.7 (44) | 27.4 (83) | <0.001 |
| **Jugular venous pressure (%(n))** | 0 (0) | 22.1 (120) | 85.1 (348) | <0.001 |
| **Hepatomegaly (%(n))** | 5.8 (27) | 13.7 (74) | 33.7 (138) | <0.001 |
| **Third heart tone (%(n))** | 6.8 (32) | 11.8 (64) | 15.9 (65) | <0.001 |
| **Systolic Blood Pressure (mmHg)** | 125.2±18.8 | 123.5±22.4 | 122.2±22.2 | 0.040 |
| **Diastolic Blood Pressure (mmHg)** | 76.3±11.5 | 75.2±13.8 | 73.4±14.3 | 0.001 |
| **Heart Rate (beats/min)** | 74.6±16.6 | 81.9±19.9 | 84.3±20.5 | <0.001 |
| **ACE-inhibitors or Angiotensin receptor blockers** |  |  |  |  |
| **ACE-inhibitors or Angiotensin receptor blockers at baseline (%(n))** | 75.4 (353) | 77.5 (420) | 70.2 (287) | 0.088 |
| Target dose at baseline(%(n)) | 20.7 (73) | 18.6 (78) | 17.1 (49) | 0.242 |
| Percentage of target dose at baseline (%) | 20 [10-50] | 20 [10-50] | 20 [0-50] | <0.001 |
| **ACE-inhibitors or Angiotensin receptor blockers at 3 months (%(n))** | 92.7 (434) | 91.1 (494) | 82.2 (336) | <0.001 |
| Target dose at 3 months (%(n)) | 28.3 (123) | 24.3 (120) | 22.9 (77) | 0.078 |
| Percentage of target dose at 3 months (%) | 50 [20-100] | 50 [20-70] | 20 [10-50] | <0.001 |
| **ACE-inhibitors or Angiotensin receptor blockers at 9 months (%(n))** | 92.1 (431) | 89.7 (486) | 79 (323) | <0.001 |
| Target dose at 9 months (%(n)) | 30.2 (130) | 27.8 (135) | 22.3 (72) | 0.018 |
| Percentage of target dose at 9 months (%) | 50 [20-100] | 50 [20-80] | 20 [10-50] | <0.001 |
| **Mineralocorticoid antagonists** |  |  |  |  |
| **MRA at baseline (%(n))** | 59.8 (280) | 56.5 (306) | 58.7 (240) | 0.698 |
| Target dose at baseline (%(n)) | 16.0 (40) | 19.3 (55) | 24.8 (56) | 0.017 |
| Percentage of target dose at baseline (%) | 50 [50-50] | 50 [50-50] | 50 [50-68.8] | 0.285 |
| **MRA at 9 months (%(n))** | 61.9 (255) | 61.2 (274) | 63.4 (189) | 0.715 |
| Target dose at 9 months (%(n)) | 16.0 (42) | 16.1 (49) | 20.4 (45) | 0.224 |
| Percentage of target dose at 9 months (%) | 50 [25-50] | 50 [25-50] | 50 [25-50] | 0.977 |
| **Loop diuretics** |  |  |  |  |
| **Loop diuretic dose at baseline** | 40 [40-75] | 40 [40-115] | 80 [40-150] | <0.001 |
| **Loop diuretic dose at 9 months** | 40 [40-60.6] | 40 [40-80] | 75 [40-120] | <0.001 |
| **Percentage loop diuretic dose change** | 0 [-50-0] | 0 [-50-0] | 0 [-60-20] | 0.011 |
| **Loop diuretic use 9 months (%)** | 88.3 (364) | 90.4 (405) | 93.3 (278) | 0.028 |
| **Laboratory** |  |  |  |  |
| **eGFR (ml/min/1.73m2)** | 64.7 [48.8-82.7] | 60.5 [47.2-77.8] | 52 [37.2-70.1] | <0.001 |
| **NT-proBNP** | 1588 [615.6-3375] | 2696 [1205-5741] | 4602 [2510.5-9321] | <0.001 |
| **Clinical Profile at 9 months** |  |  |  |  |
| **Oedema (%(n))** | 2.7 (10) | 8.8 (34) | 12.6 (31) | <0.001 |
| **Rales > 1/3 up lung fields (%(n))** | 6.9 (2) | 6.7 (3) | 3.6 (1) | 0.596 |
| **Jugular venous pressure (%(n))** | 4.9 (16) | 7.3 (25) | 15.5 (34) | <0.001 |
| **Hepatomegaly (%(n))** | 3.1 (12) | 5.4 (23) | 10.3 (28) | <0.001 |
| **Orthopnoea (%(n))** | 4 (16) | 8.8 (39) | 13.6 (39) | <0.001 |
| **Third heart tone (%(n))** | 2.3 (9) | 4.4 (19) | 4 (11) | 0.204 |
| **Systolic Blood Pressure (mmHg)** | 125.6±20.1 | 123±20.5 | 123.2±19.7 | 0.136 |
| **Diastolic Blood Pressure (mmHg)** | 75.3±11.5 | 73.9±12 | 73.7±12.9 | 0.088 |
| **Heart Rate (beats/min)** | 70.8±14.8 | 71.7±13.9 | 74.8±14.9 | 0.001 |
| **NYHA class (%(n))** |  |  |  | <0.001 |
| **I** | 16 (75) | 11.1 (60) | 8.3 (34) |  |
| **II** | 54.1 (253) | 47 (255) | 35.7 (146) |  |
| **III** | 14.5 (68) | 20.8 (113) | 22.5 (92) |  |
| **IV** | 0 (0) | 1.7 (9) | 0.7 (3) |  |
| **Clinical congestion score** | 1 [1-1] | 1 [1-2] | 1 [1-3] | <0.001 |

***Abbreviations:*** BMI: body mass index, eGFR: estimated glomerular filtration rate, LVEF: left ventricular ejection fraction, MRA: mineralocorticoid antagonists, NT-proBNP: n terminal pro blood natriuretic peptide, NYHA: New York Heart Association

***Supplementary table 7: Groups based on change in congestion score and loop diuretic dose at 9 months***

|  | **Decrease in clinical congestion score from baseline to 9 months** | | |  | **No change/increase in clinical congestion score from baseline to 9 months** | | |  |
| --- | --- | --- | --- | --- | --- | --- | --- | --- |
|  | **Low dose LD** | **Medium**  **dose LD** | **High dose LD** | ***P-trend*** | **Low dose LD** | **Medium**  **dose LD** | **High dose LD** | ***P-trend*** |
| **N =** | 87 | 200 | 161 |  | 101 | 162 | 141 |  |
| **Change in congestion score** | -1 [-1.3--0.67] | -1.3 [-1.67--1.0] | -1.3 [-2.0--1.0] |  | 0 [0-0] | 0 [0-0] | 0 [0-0.3] |  |
| **Loop diuretic dose at 9 months** | 20 [0-20] | 40 [40-40] | 120 [80-200] |  | 20 [0-25] | 40 [40-40] | 125 [80-175] |  |
| **ACE-inhibitors or Angiotensin receptor blockers** |  |  |  |  |  |  |  |  |
| **ACE-inhibitors or Angiotensin receptor blockers at baseline (%(n))** | 80.5 (70) | 75 (150) | 70.8 (114) | 0.095 | 77.2 (78) | 87 (141) | 80.9 (114) | 0.607 |
| Target dose at baseline (%(n)) | 12.9 (9) | 21.3 (32) | 20.2 (23) | 0.291 | 10.3 (8) | 28.4 (40) | 10.5 (12) | 0.638 |
| Percentage of target dose at baseline (%) | 20 [10-50] | 20 [10-50] | 20 [0-50] | 0.911 | 20 [10-50] | 50 [20-70] | 20 [10-50] | 0.352 |
| **ACE-inhibitors or Angiotensin receptor blockers at 3 months (%(n))** | 92 (80) | 94 (188) | 85.1 (137) | 0.029 | 91.1 (92) | 97.5 (158) | 92.9 (131) | 0.722 |
| Target dose at 3 months (%(n)) | 23.8 (19) | 26.1 (49) | 21.9 (30) | 0.653 | 31.5 (29) | 34.2 (54) | 22.1 (29) | 0.089 |
| Percentage of target dose at 3 months (%) | 50 [20-80] | 50 [20-80] | 40 [10-50] | 0.085 | 50 [20-100] | 50 [20-100] | 40 [20-60] | 0.068 |
| **ACE-inhibitors or Angiotensin receptor blockers at 9 months (%(n))** | 90.8 (79) | 93.5 (187) | 84.5 (136) | 0.044 | 93.1 (94) | 97.5 (158) | 87.2 (123) | 0.041 |
| Target dose at 9 months (%(n)) | 27.8 (22) | 31 (58) | 25 (34) | 0.522 | 35.1 (33) | 32.9 (52) | 25.2 (31) | 0.106 |
| Percentage of target dose at 9 months (%) | 50 [20-100] | 50 [20-100] | 30 [10-60] | 0.034 | 50 [20-100] | 50 [20-100] | 20 [10-60] | 0.001 |
| **Mineralocorticoid antagonists** |  |  |  |  |  |  |  |  |
| **MRA at baseline (%(n))** | 54.0 (47) | 54.0 (108) | 67.1 (108) | 0.020 | 43.6 (44) | 65.4 (106) | 66 (93) | 0.001 |
| Target dose at baseline (%(n)) | 7.3 (3) | 15 (16) | 29.5 (28) | 0.001 | 8.8 (3) | 10.4 (11) | 21.0 (17) | 0.038 |
| Percentage of target dose at baseline (%) | 50 [50-50] | 50 [50-50] | 50 [50-100] | 0.003 | 50 [50-50] | 50 [50-50] | 50 [50-50] | 0.027 |
| **MRA at 9 months (%(n))** | 47.7 (41) | 65.0 (130) | 77.6 (125) | <0.001 | 39.6 (40) | 72.8 (118) | 62.4 (88) | 0.002 |
| Target dose at 9 months (%(n)) | 13.7 (7) | 19.1 (27) | 21.6 (25) | 0.256 | 9.3 (4) | 13.7 (17) | 16.5 (15) | 0.269 |
| Percentage of target dose at 9 months (%) | 50 [0-50] | 50 [50-50] | 50 [50-50] | 0.013 | 25 [0-50] | 50 [50-50] | 50 [25-50] | 0.129 |
| **Loop diuretics** |  |  |  |  |  |  |  |  |
| **Loop diuretic dose at baseline** | 40 [40-60] | 40 [40-80] | 100 [40-175] | <0.001 | 40 [25-40] | 40 [40-40] | 80 [40-150] | <0.001 |
| **Percentage loop diuretic dose change** | -75 [-100--50] | 0 [-50-0] | 0 [-16.7-100] | <0.001 | -50 [-100-0] | 0 [0-0] | 0 [0-100] | <0.001 |
| **Clinical Profile at 9 months** |  |  |  |  |  |  |  |  |
| **Systolic Blood Pressure (mmHg)** | 129.3±22.7 | 123.8±19.4 | 121.5±19 | 0.005 | 126.9±20.7 | 127.1±19.7 | 120.8±21.2 | 0.015 |
| **Diastolic Blood Pressure (mmHg)** | 75.4±13.1 | 75.2±12.6 | 72.7±11.5 | 0.064 | 75.5±11.7 | 76.7±11.2 | 72.5±11.4 | 0.026 |
| **Heart Rate (beats/min)** | 71.7±14.1 | 73±13.2 | 73.6±12.9 | 0.296 | 71.1±14.8 | 71.2±13.8 | 72.4±15.5 | 0.474 |
| **NYHA class (%(n))** |  |  |  | <0.001 |  |  |  | <0.001 |
| **I** | 27.6 (24) | 14 (28) | 8.1 (13) |  | 33.7 (34) | 9.3 (15) | 7.8 (11) |  |
| **II** | 59.8 (52) | 64 (128) | 54 (87) |  | 51.5 (52) | 74.1 (120) | 43.3 (61) |  |
| **III** | 11.5 (10) | 19.5 (39) | 35.4 (57) |  | 13.9 (14) | 14.8 (24) | 44.7 (63) |  |
| **IV** | 0 (0) | 0.5 (1) | 0.6 (1) |  | 0 (0) | 1.9 (3) | 3.5 (5) |  |
| **Clinical congestion score** | 0 [0-0] | 0 [0-0] | 0 [0-0.3] | <0.001 | 0 [0-0] | 0 [0-0] | 0.3 [0-1] | <0.001 |
| **Haematocrit (%)** | 39.2 [36.0-43.2] | 40.1 [37.5-43.0] | 40.0 [36.0-42.9] | 0.786 | 41.5 [38.5-44.0] | 40.5 [38.5-43.6] | 38.9 [35.0-42.0] | 0.002 |
| **BUN/creatinine ratio** | 12.2 [6.2-16.4] | 11.8 -8.3-14.8] | 9.6 [5.4-12.5] | 0.002 | 9.8 [5.9-14.0] | 12.1 [9.9-15.0] | 9.8 [6.2-12.8] | 0.450 |

***Abbreviations:*** ACE: angiotensin converting enzyme, BUN: blood urea nitrogen, NYHA: New York Heart Association, LD: loop diuretics

***Supplementary table 8. Cox regression analysis for the combined endpoint of all-cause mortality and heart failure hospitalization***

|  | **Combined endpoint** | |  |  |
| --- | --- | --- | --- | --- |
|  | Univariable |  | Multivariable^#^ |  |
|  | HR (95% CI) | P-value | HR (95% CI) | P-value |
| **Log loop diuretic dose** | 1.71 (1.58-1.85) | <0.001 | 1.16 (1.06-1.27) | 0.002 |
|  |  |  |  |  |
| **Low dose (<80 mg of furosemide)** | Ref | Ref | Ref | Ref |
| **High dose (≥80 mg of furosemide)** | 1.92 (1.69-2.19) | <0.001 | 1.25 (1.09-1.42) | <0.001 |
|  |  |  |  |  |
| **Low dose & ≥50% of ACEi target dose** | Ref | Ref | Ref | Ref |
| **Low dose & <50% of ACEi target dose** | 1.39 (1.12-1.73) | 0.003 | 1.04 (0.83-1.30) | 0.726 |
| **High dose & <50% of ACEi target dose** | 2.82 (2.28-3.48) | <0.001 | 1.38 (1.09-1.73) | 0.006 |
| **High dose & ≥50% of ACEi target dose** | 1.71 (1.36-2.15) | <0.001 | 1.22 (0.97-1.55) | 0.090 |

^#^ adjusted for the BIOSTAT risk model for the combined endpoint (age, HF hospitalization in previous year, systolic blood pressure, log NT-proBNP, hemoglobin, high-density lipoprotein, sodium, and beta-blocker use at baseline)

*Abbreviations:* ACEi: angiotensin converting enzyme inhibitors, CI: confidence interval, HR: hazard ratio.
